# Vulcanite Plates

**Published:** 1885-02

**Authors:** 


### VULCANITE PLATES.

The advocates of rubber dentures are always upon the defensive. Urge as one may their good qualities, there yet sticks in the mind of every unprejudiced dentist the fact that caoutchouc is *not* the best material to be placed in continuous contact with soft tissues, especially the mucous membrane of the mouth. There probably is not a dentist using the base who does recognize this fact, and deplore it. Various means of overcoming this incompatibility have been devised, heretofore with but poor success. We have, however, lately been experimenting with "Vulcanizable Gold," advertised in this number, and are of the opinion that a long step towards solving the problem has been made. It is easily applied to the palatal portion of a plate, and it will not peel off, while it entirely prevents the contact of the rubber with the oral vault. The plate is very much improved in appearance and value, and certainly it must prove more tolerable to the tissues. We think the makers of the better class of rubber plates would do well to try it.

